# Polyfluoroalkyl Chemicals and Menopause among Women 20–65 Years of Age (NHANES)

**DOI:** 10.1289/ehp.1306707

**Published:** 2013-11-26

**Authors:** Kyla W. Taylor, Kate Hoffman, Kristina A. Thayer, Julie L. Daniels

**Affiliations:** 1 Office of Health Assessment and Translation, Division of the National Toxicology Program, National Institute of Environmental Health Sciences, National Institutes of Health, Department of Health and Human Services, Research Triangle Park, North Carolina, USA; 2 University of North Carolina Gillings School of Global Public Health, Chapel Hill, North Carolina, USA

## Abstract

Background: Polyfluoroalkyl chemicals (PFCs) such as perfluorooctane sulfonate (PFOS) and perfluorooctanoate (PFOA) have been associated with early menopause. However, previous cross-sectional studies have lacked adequate data to investigate possible reverse causality (i.e., higher serum concentrations due to decreased excretion after menopause).

Objectives: We investigated the association between PFOS, PFOA, perfluorononanoate (PFNA), and perfluorohexane sulfonate (PFHxS) and age at natural menopause among women 20–65 years of age in NHANES (National Health and Nutrition Examination Survey).

Methods: We used proportional hazard models to estimate hazard ratios (HRs) for the onset of natural menopause as a function of age and serum PFC levels, and to investigate reverse causation by estimating associations between PFC levels and the rate of hysterectomy. We also used multivariable linear regression to determine whether time since menopause predicted serum PFC levels.

Results: After adjusting for age at survey, race/ethnicity, education, ever smoking, and parity, women with higher levels of PFCs had earlier menopause than did women with the lowest PFC levels. We observed a monotonic association with PFHxS: The HR was 1.42 (95% CI: 1.08, 1.87) for serum concentrations in tertile 2 versus tertile 1, and 1.70 (95% CI: 1.36, 2.12) for tertile 3 versus tertile 1. We also found evidence of reverse causation: PFCs were positively associated with rate of hysterectomy, and time since natural menopause was positively associated with serum PFCs.

Conclusions: Our ﬁndings suggest a positive association between PFCs and menopause; however, at least part of the association may be due to reverse causation. Regardless of underlying cause, women appear to have higher PFC concentrations after menopause.

Citation: Taylor KW, Hoffman K, Thayer KA, Daniels JL. 2014. Polyfluoroalkyl chemicals and menopause among women 20–65 years of age (NHANES). Environ Health Perspect 122:145–150; http://dx.doi.org/10.1289/ehp.1306707

## Introduction

Polyfluoroalkyl chemicals (PFCs) are man-made compounds that have been used in a number of common consumer and industrial products such as food containers; stain- and water-resistant protection for clothing, furniture, and carpets; paints; fire-fighting foam; and photographic emulsifiers (Lau et al. 2007). PFCs are ubiquitously present and persistent in the environment (Lau et al. 2007), and although there are demographic, geographic, and temporal differences, exposures in the general population are widespread. Four PFC analytes—perfluorooctane sulfonate (PFOS), perfluorooctanoate (PFOA), perfluorononanoate (PFNA), and perfluorohexane sulfonate (PFHxS)—are commonly detected in humans (Calafat et al. 2006; Fromme 2007; Kato et al. 2011). Unlike traditional persistent organic pollutants, which are lipophilic and stored primarily in fat tissue, PFOS and PFOA are both lipophobic and hydrophobic. After absorption, they persist in the body by forming chemical bonds to proteins in serum, rather than accumulating in lipids (Jones et al. 2003; Organisation for Economic Co-operation and Development 2002). Serum levels of PFCs reflect long-term exposures to these contaminants [U.S. Environmental Protection Agency (EPA) 2012b], with estimated geometric mean half-lives of 7.3 years (95% CI: 5.8, 9.2) for PFHxS, 4.8 years (95% CI: 4.0, 5.8) for PFOS, and 3.5 years (95% CI: 3.0, 4.1) for PFOA (Olsen et al. 2007). However, in a more recent study, Bartell et al. (2010) estimated a shorter median half-life for serum PFOA (2.3 years; 95% CI: 2.1, 2.4).

PFCs are potential endocrine disruptors, and effects of PFOS and PFOA on endocrine function have been reported in animal studies (Jensen and Leffers 2008; Zhao et al. 2010). Less is known about associations between PFCs and human endocrine function. Melzer et al. (2010) reported that higher concentrations of serum PFOA and PFOS were associated with current thyroid disease based on NHANES (National Health and Nutrition Examination Survey) data from 1999–2000, 2003–2004, and 2005–2006. In addition, in a population-based cohort of adolescents and young adults in Taiwan, Lin et al. (2013) observed a positive association between serum levels of PFNA and serum levels of thyroxine (T_4_). Yet, other studies have reported no association between PFOS or PFOA levels and thyroid function. These include occupational studies with high levels of exposure (Olsen et al. 2003; Olsen and Zobel 2007); a study of residents of a water district in southeastern Ohio, where there is significant environmental exposure to PFOA (Emmett 2006); studies of populations in Korea (Ji et al. 2012) and China (Lin et al. 2013); a study of Inuit adults (Dallaire et al. 2009); and studies of pregnant women (Chan et al. 2011; Inoue et al. 2004). In the United States, one of the largest efforts to investigate the impact of exposures to PFCs was initiated by the C8 Science Panel, which was created as part of a settlement agreement stemming from PFOA (or C8) contamination of drinking water in six water districts in two states near the DuPont Washington Works facility near Parkersburg, West Virginia (Frisbee et al. 2009). In this exposed population, high environmental levels of PFOA in water were associated with delayed onset of puberty in girls (Lopez-Espinosa et al. 2011) as well as earlier menopause (Knox et al. 2011). Knox et al. (2011) found that serum PFOS and PFOA were significantly higher (*p* < 0.0001) in women 40–55 years of age who had a hysterectomy compared with women who had not. However, because the authors did not observe the timing of PFC exposure relative to menopause, causal inference is limited. One noncausal explanation for an association between PFCs and natural menopause is that elimination of PFCs via the loss of menstrual blood and tissue might result in lower serum levels in menstruating women than in postmenopausal women (Knox et al. 2011).

We investigated associations between multiple PFCs (PFOS, PFOA, PFNA, and PFHxS) and age at natural and surgically induced (hysterectomy) menopause using NHANES data. NHANES collected information regarding the age at which women experienced menopause, which allowed us to investigate the relationship between serum levels and time since menopause and the possibility of reverse causality.

## Methods

*Study population*. NHANES is a nationally representative, cross-sectional survey of about 5,000 persons each year, with survey participants located in counties across the United States. The survey uses in-home interviews and physical examinations in a mobile examination unit to collect data on demographics, behavioral and environmental risk factors, and health status [Centers for Disease Control and Prevention (CDC) 2012]. Written consent was obtained from all NHANES participants after approval by the NCHS (National Center for Health Statistics) Research Ethics Review Board (CDC 2012). Details regarding interview, examination, and sample collection protocols have been described previously (CDC 2013a). Because the present study is based on previously collected data that have been deidentified, the University of North Carolina Institutional Review Board determined the study to be exempt from review.

*PFC measurement*. The National Center for Environmental Health analyzed individual serum PFC levels in five NHANES sample waves: 1999–2000, 2003–2004, 2005–2006, 2007–2008, and 2009–2010. In each wave, a random one-third subset of participants ≥ 12 years of age was selected for assessment of 12 PFCs (CDC 2009). Detailed analytic methods have been described previously (Calafat et al. 2007a, 2007b; Kato et al. 2011). Briefly, serum samples were analyzed using automated solid-phase extraction coupled to reverse-phase high-performance liquid chromatography/tandem mass spectrometry. The laboratory methods and comprehensive quality control system were consistent across each NHANES wave, and documentation for each wave is available online (CDC 2013b). We examined PFOS, PFOA, PFNA, and PFHxS in relation to menopausal status because these compounds were detected in > 95% of samples compared with other PFCs, which were detected infrequently (Calafat et al. 2007a, 2007b; Kato et al. 2011). Any participant with a serum PFC concentration below the limit of detection (LOD) was assigned a serum level of the LOD divided by the square root of 2 (Calafat et al. 2007a, 2007b; Kato et al. 2011). Serum PFC concentrations were categorized into tertiles based on distributions among all women in the study sample. We considered other categorizations, including quartiles and quintiles, but results were similar regardless of the categorization used (results not shown). We chose to categorize by tertile to increase the stability of our estimates.

**Figure 1:**
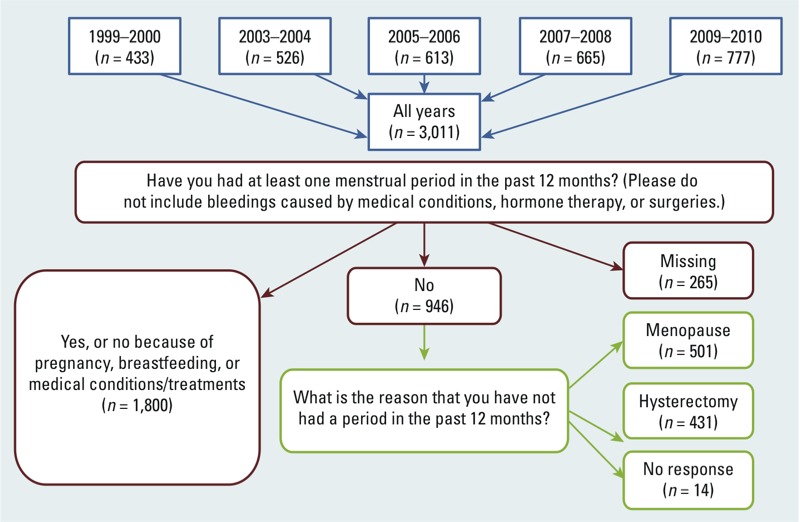
Study population of women 20–65 years of age who had PFC serum measurements: NHANES years 1999–2000, 2003–2004, 2005–2006, 2007–2008, and 2009–2010.

*Menopausal status*. Women ≥ 18 years of age completed a reproductive health questionnaire. They were asked “Have you had at least one menstrual period in the past 12 months? (Please do not include bleedings caused by medical conditions, hormone therapy, or surgeries.).” Women who answered “no” were subsequently asked “What is the reason that you have not had a period in the past 12 months?” We classified women as premenopausal if they answered “yes” to the first question or answered “no” to the first question but indicated the reason as pregnancy, breastfeeding, irregular periods, or medical conditions/treatments (*n* = 1,800). We classified women as postmenopausal if they answered “no” to the first question and indicated the reason to be natural menopause (*n* = 501) or hysterectomy (*n* = 431). We excluded from all analyses 265 women with no information on their menopausal status and 14 who answered that they had not had their period in the last 12 months but did not state why (Figure 1). The distribution of demographic characteristics and PFC levels among these women were similar to those of the combined larger sample, except that most women were missing data for parity. For women who reported having gone through menopause, age at occurrence was recorded. We calculated time since menopause by subtracting the age at which postmenopausal women reported having their last menstrual period from their age at the time of survey.

*Statistical analyses*. We used proportional hazard modeling to estimate hazard rate [hazard ratios (HRs)] of the onset of natural menopause as a function of age and serum PFC levels. Premenopausal women were censored at their age at the time of the survey. Analyses were performed in SAS (version 9.2; SAS Institute Inc., Cary, NC) using the Proc SURVEYPHREG procedure, which accounts for stratification and clustering within primary sampling units used to select the NHANES sample. Rather than using NHANES sample weights, we adjusted all models for covariates related to the NHANES sample selection procedure (age and race/ethnicity), a method that balances the trade-off between efficiency and bias (Graubard and Korn 1999; Korn and Graubard 1991). To determine the robustness of our results, we also conducted analyses using the NHANES sample weights. The inclusion of weights made no appreciable difference in the magnitude or precision of the associations (results not shown); therefore, we present the unweighted analyses.

**Figure 2:**
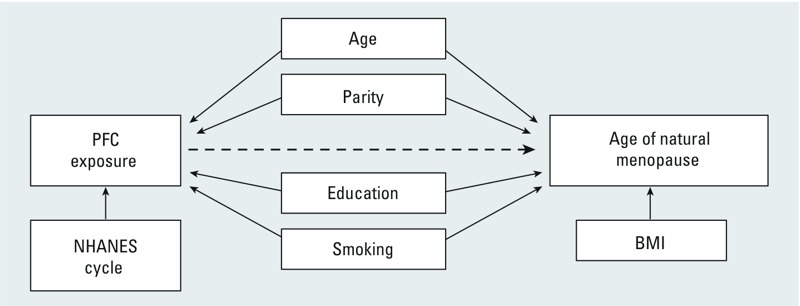
Directed acyclic graph of association between PFC exposure and age of natural menopause.

Covariates. We considered a number of covariates as possible confounders in the association between PFCs and menopause. We used information on the association between other persistent organic pollutants and menopause to develop a directed acyclic graph (Weng et al. 2009) that inferred the following confounders: age, race, parity, education, and smoking (Figure 2) (Cooper et al. 2002; Knox et al. 2011). We did not adjust for body mass index (BMI; body weight in kilograms divided by height in meters squared) for several reasons. Steenland et al. (2009) reported an association between serum PFC levels and BMI, suggesting that BMI could be a potential intermediate on the causal pathway between PFC exposure and menopause. High BMI has also been associated with later onset of natural menopause (Akahoshi 2002). However, in a study using data from NHANES, Nelson et al. (2010) found no consistent association between serum PFC levels and BMI; thus, BMI did not meet criteria as a potential confounder (Greenland and Robins 1986). Because BMI generally increases after menopause, BMI reported at the time of the survey may not reflect BMI at the time of menopause. Therefore, we examined whether inclusion of BMI in our model would appreciably alter the association between PFCs and menopause (HR change > 10%) and found that it did not (results not shown). We also considered adjustment for NHANES wave. However, because sample wave was strongly associated with serum PFC levels but not age at menopause, it was not included as a covariate. Age at the time of interview was modeled as a continuous variable. Race/ethnicity, education, smoking, and parity were modeled as categorical variables (Table 1).

**Table 1:** Demographic characteristics of women 20–65 years of age who either had their period (premenopausal), had experienced menopause, or had experienced hysterectomy: NHANES 1999–2000, 2003–2004, 2005–2006, 2007–2008, and 2009–2010.^*a*^

Characteristic	Premenopause	Menopause	Hysterectomy
Population (*n*)	1,800	501	431
Age at interview (years; median ± SD)	34 ± 9.61	58 ± 5.25	55 ± 8.46
Age at last period (years; median ± SD)		49 ± 4.67	38 ± 7.66
NHANES cycle
1999–2000	282 (15.67)	67 (13.37)	48 (11.14)
2003–2004	298 (16.56)	84 (16.77)	98 (22.74)
2005–2006	403 (22.39)	83 (16.57)	75 (17.40)
2007–2008	389 (21.61)	125 (24.95)	110 (25.52)
2009–2010	428 (23.78)	142 (28.34)	100 (23.20)
Race/ethnicity
White non-Hispanic	790 (43.89)	225 (44.91)	209 (48.49)
Black non-Hispanic	345 (19.17)	96 (19.16)	101 (23.43)
Mexican American	433 (24.06)	115 (22.95)	65 (15.08)
Other Hispanic	159 (8.83)	43 (8.58)	38 (8.82)
Other (including multi­racial)	73 (4.06)	22 (4.39)	18 (4.18)
Education (years)
< 12	440 (24.47)	158 (31.54)	112 (25.99)
12	359 (19.97)	100 (19.96)	127 (29.47)
> 12	999 (55.56)	243 (48.50)	192 (44.55)
Missing	2	0	0
BMI (kg/m^2^)
Underweight (< 18.5)	46 (2.56)	6 (1.21)	4 (0.94)
Normal weight (18.5 to < 25)	569 (31.74)	129 (26.01)	72 (16.98)
Overweight (25 to < 30)	516 (28.76)	140 (28.23)	123 (29.01)
Obese (> 30)	663 (36.96)	221 (44.56)	225 (53.07)
Missing	6	5	7
Ever smoked
No	1,188 (66.00)	270 (53.89)	230 (53.36)
Yes	612 (34.00)	231 (46.11)	201 (46.64)
Missing	0	0	0
Parity (no. of live births)
0	388 (23.08)	62 (13.11)	39 (9.24)
1	339 (20.17)	66 (14.00)	60 (14.22)
> 1	954 (56.75)	344 (72.89)	323 (76.54)
Missing	119	29	9
PFC exposure [ng/mL; median (T1, T3)]
PFOS	10.3 (6.0, 17.0)	14.03 (8.80, 23.9)	17.50 (10.6, 29.4)
PFOA	2.70 (1.80, 4.20)	3.80 (2.50, 5.30)	4.20 (2.90, 5.90)
PFNA	0.90 (0.60, 1.40)	1.20 (0.80, 1.80)	1.30 (0.80, 2.10)
PFHxS	1.00 (0.60, 1.80)	1.50 (0.90, 2.60)	1.70 (1.10, 3.10)
T, tertile. Values are *n* (%) except where indicated. ^***a***^Women with unknown menopausal status were excluded.			

Reverse causation. PFCs may be excreted in blood and tissue during menstruation (Knox et al. 2011); therefore, women who are no longer menstruating may have higher levels of PFCs because they lack that elimination pathway. Consequently, any association between serum PFC levels and rate of natural menopause may be the result of reverse causation. We investigated the potential for reverse causation in two ways. First, we used proportional hazard models to examine the association between PFC levels and rate of hysterectomy. Premenopausal women were censored at their age at interview, and postmenopausal women were censored at the age of menopause. If we assume that the reasons for hysterectomy are not related to PFC exposure, an observed positive association between PFC levels and hysterectomy suggests reverse causation. Conversely, if there is no reverse causation, we would expect to see no association between PFC level and hysterectomy. Second, we investigated whether the rate of natural menopause predicts PFC levels; if the absence of menstrual blood and tissue loss explains the association between PFCs and self-reported natural menopause, we would expect women who menstruated more recently to have lower serum levels of PFCs. We used generalized additive models (GAMs) to examine the shape of the relationship between years since natural menopause and PFCs among postmenopausal women and determined that a linear approximation appropriately represented the shape of the relationship (results not shown). We report results from linear regression models in SAS Proc SURVEYREG. Statistical significance was defined as *p* = 0.05.

## Results

Among the 2,732 women with both PFC measurements and menstrual status data, 65.9% (*n* = 1,800) were premenopausal, 18.3% (*n* = 501) had completed natural menopause, and 15.7% (*n* = 431) had hysterectomies (Table 1). At the time of the survey, premenopausal women were generally younger (median age, 34 years), and women who had gone through natural menopause were the oldest (median age, 58 years). The median age at last period among women who had completed natural menopause was 49 years. We detected PFOS, PFOA, PFHxS, and PFNA in at least 95% of serum samples, similar to previous reports from the larger NHANES population (Calafat et al. 2007a, 2007b; Kato et al. 2009). The premenopausal group had the lowest median PFC levels, whereas the posthysterectomy group had the highest median levels (Table 1). For women in our study, serum collection and completion of reproductive health questionnaires took place a median of 7.5 years after natural menopause [interquartile range (IQR), 8 years] and a median of 14 years after hysterectomy (IQR, 16.5 years). In addition, PFC measurements were available for 265 women who lacked data on menstrual status and for 14 who indicated they had not had a period in the past year but did not report the reason.

**Figure 3:**
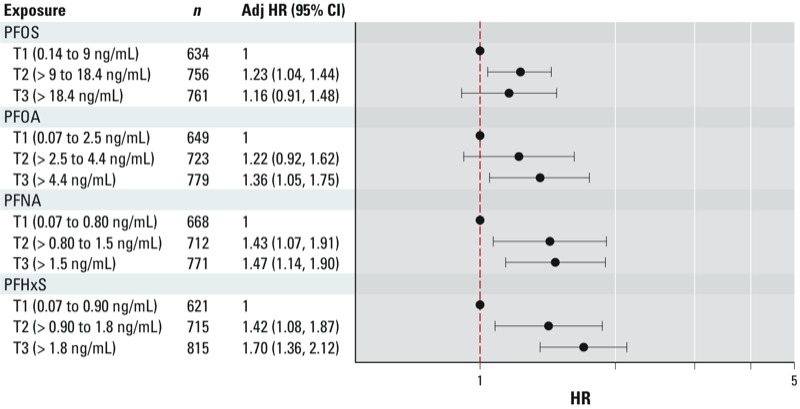
Adjusted (Adj) HRs and 95% CIs for menopause in association with tertiles (T) of serum PFCs among women 20–65 years of age: NHANES 1999–2000, 2003–2004, 2005, 2006, 2007–2008, and 2009–2010. Data are based on the proportional hazards model for age at menopause, censoring at interview age if still menstruating, and eliminating all cases of hysterectomy. HRs are adjusted for age at interview, race/ethnicity, education, smoking, and parity.

After adjusting for age at interview, race/ethnicity, education, smoking status, and parity, women with higher serum levels of PFCs (tertiles 2 and 3) consistently had higher rates of menopause than women in tertile 1 (Figure 3). There appeared to be a monotonic dose–response association of PFOA, PFNA, and PFHxS with menopause. The adjusted HRs for women with the highest serum levels of PFOA, PFNA, and PFHxS (tertile 3 compared with tertile 1) were 1.36 (95% CI: 1.05, 1.75), 1.47 (95% CI: 1.14, 1.90), and 1.70 (95% CI: 1.36, 2.12), respectively. The adjusted HRs for tertile 2 versus tertile 1 were 1.22 (95% CI: 0.92, 1.62) for PFOA, 1.43 (95% CI: 1.07, 1.91) for PFNA, and 1.42 (95% CI: 1.08, 1.87) for PFHxS. For serum PFOS, adjusted HRs were higher in tertile 2 than in tertile 3.

**Figure 4:**
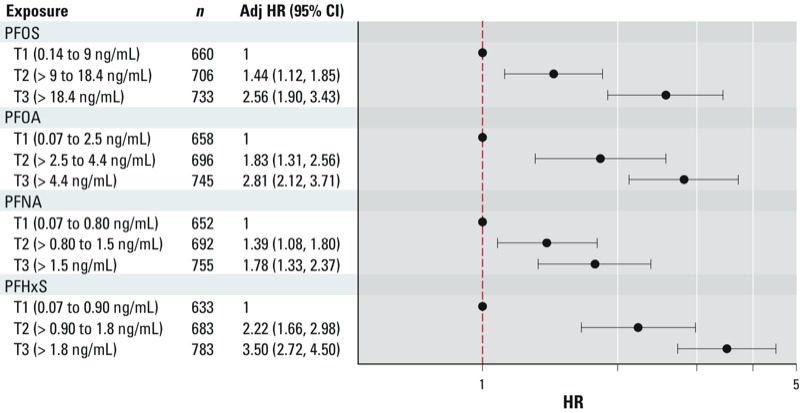
Adjusted (Adj) HRs and 95% CIs for hysterectomy in association with tertiles (T) of serum PFCs among women: 20–65 years of age NHANES 1999–2000, 2003–2004, 2005, 2006, 2007–2008, and 2009–2010. Data are based on the proportional hazards model for age at hysterectomy, censoring at interview age if still menstruating, and eliminating all cases of menopause. HRs are adjusted for age at interview, race/ethnicity, education, smoking, and parity.

We observed robust, positive dose–response associations for all four PFCs and hysterectomy (Figure 4). PFHxS was most strongly associated with the rate of hysterectomy (adjusted HR = 3.50; 95% CI: 2.72, 4.50) in tertile 3. Although the HRs for PFOS and PFOA are lower than those for PFHxS, the monotonic pattern of increasing HRs for PFOS and PFOA appears fairly similar to that of PFHxS. Using linear regression models, we found that levels of all four PFCs increased with each additional year since natural menopause (Table 2).

**Table 2:** Adjusted β (95% CI)^*a*^ for the change in serum PFC concentrations (ng/mL) associated with a 1-year increase in the time between natural menopause and sample collection among naturally postmenopausal women (*n* = 501): NHANES 1999–2000, 2003–2004, 2005–2006, 2007–2008, and 2009–2010.

PFC	β (95% CI)^*a*^
PFOS	0.23 (–0.16, 0.48)
PFOA	0.07 (0.013, 0.13)
PFNA	0.02 (0.002, 0.042)
PFHxS	0.023 (–0.019, 0.065)
^***a***^Adjusted for age at time of survey, race/ethnicity, education, smoking, and parity.	

## Discussion

Higher body burdens of PFCs were associated with earlier onset of natural menopause. Associations were strongest between serum levels of PFNA and PFHxS and the rate of natural menopause. PFNA and PFHxS have not been studied previously with respect to menopause, which is of concern because PFNA and PFHxS have not declined over time in the same manner as PFOA and PFOS (Andersen et al. 2008; Calafat et al. 2007b; Kato et al. 2011); geometric mean serum levels of PFNA are increasing (e.g., from 0.55 to 1.49 ng/mL between survey years 1999–2000 and 2007–2008), and serum levels of PFHxS increased in 2007–2008 compared with previous years (Kato et al. 2011). Results of the present study show positive associations between PFOS and PFOA and earlier menopause, consistent with previous reports in the literature (Knox et al. 2011). As we anticipated, serum levels of PFOA were lower in our sample (median, 3.8 ng/mL) compared with the C8 Health Study (medians of 17.6 ng/mL in women 18 to ≤ 42 years of age and 23.4 ng/mL in women > 42 and ≤ 51 years of age) (Knox et al. 2011), which had high levels of PFOA due to industrial contamination. Despite lower levels, we also observed a positive association between PFOA and the rate of natural menopause.

Although we observed associations for all PFCs assessed, we cannot rule out the possibility that associations are driven by a single congener, because some PFCs in sera are correlated. Using NHANES data, Calafat et al. (2007b) found statistically significant correlations (*p* < 0.001) between the log-transformed concentrations of PFOS and PFOA [Pearson correlation coefficient (*r*) = 0.66], PFHxs (*r* = 0.56), and PFNA (*r* = 0.50). The correlation coefficient between the log-transformed concentrations of PFOA and PFHxS was 0.46; between PFOA and PFNA, *r* = 0.55. In the present study, Spearman correlations ranged from 0.19 between PFOS and PFNA to 0.65 between PFOS and PFOA (*p* < 0.001 for all correlations). We considered the use of a total (summed) exposure measure of all four PFC congeners; however, serum levels of PFOS were much higher than those of PFOA, PFHxS, and PFNA, suggesting that the combined analysis would disproportionately reflect PFOS levels.

Early menopause is associated with a number of adverse health impacts. For example, results from a meta-analysis demonstrated that menopause before 50 years of age was associated with a 25% increased risk of cardiovascular disease (Atsma et al. 2006) and menopause before age 46 has been associated with increased risk of coronary heart disease and stroke (Lisabeth et al. 2009; Wellons et al. 2012). If PFC levels are predictors of earlier menopause, exposure may also be responsible for increased risk of other serious health outcomes (e.g., cardiovascular disease and stroke).

Our examination of reverse causality indicated positive associations between all four of the PFCs we examined and the rate of hysterectomy, and showed that these PFC levels increased with time since natural menopause. Taken together, the results of these two additional analyses suggest that the association between PFCs and menopause may reflect the accumulation of PFCs among women who were not excreting them through menstruation. However, because of the cross-sectional natural of our data, we cannot confirm the direction of these associations. Prospective human studies evaluating the onset of menopause are necessary to better assess potential causality.

Using data from the large, U.S. representative NHANES sample allowed us to explore the association between PFCs and the hazard of natural menopause while adjusting for potential confounding by a number of variables. Unlike previous analyses of PFCs and natural menopause, NHANES collected information on the time since natural menopause and surgical hysterectomy, which allowed us to address the potential for reverse causality. The cross-sectional nature of data collection does not allow us to establish temporality because menopause status, age at menopause, and PFC measurements were taken at the same time. PFC measurements were based on a single serum sample. Any misclassification from single measures would tend to decrease power and underestimate the real strengths of association (Pearce et al. 2007). Although a single sample may be more reliable for compounds with long half-lives, samples collected at several time points would be more accurate for classifying exposure in future studies (Melzer et al. 2010).

## Conclusions

The consistency and robustness of our ﬁndings suggest a relationship between PFCs and menopause, although the underlying mechanism of that association remains unknown. In these cross-sectional data, it is not clear whether the association observed between PFCs and menopause is causal, if results are due to noncausal influences such as biases from confounding or misclassification, or if results are due to accumulation of PFCs after menopause. Regardless of the underlying cause, women appear to accumulate PFCs more rapidly after they are no longer menstruating. These results, along with the ubiquitous nature of exposure and persistence of PFCs in the environment, support the need for continued monitoring of serum levels in the general population as well as further studies of the reproductive health effects of PFCs.
